# Distinguishing the papilla of Vater during biliary cannulation using texture and color enhancement imaging: A pilot study

**DOI:** 10.1002/deo2.125

**Published:** 2022-05-15

**Authors:** Kazuya Miyaguchi, Masafumi Mizuide, Yuki Tanisaka, Akashi Fujita, Ryuhei Jinushi, Katsuda Hiromune, Tomoya Ogawa, Yoichi Saito, Tomoaki Tashima, Yumi Mashimo, Hiroyuki Imaeda, Shomei Ryozawa

**Affiliations:** ^1^ Department of Gastroenterology Saitama Medical University International Medical Center Saitama Japan; ^2^ Department of Gastroenterology Saitama Medical University Saitama Japan

**Keywords:** biliary cannulation, endoscopists, papilla of Vater, texture and color enhancement imaging, white light imaging

## Abstract

**Objectives:**

Understanding the exact morphology of the bile duct opening is important for determining the success of bile duct cannulation. Texture and color enhancement imaging (TXI) has been reported to enhance slight changes in color tone and structure that are difficult to see with white light imaging. This study investigated whether TXI mode1 could improve papillary recognition by trainees inexperienced in endoscopic retrograde cholangiopancreatography.

**Methods:**

We included 31 patients with naive papilla of Vater at a single institution in the study. Trainee endoscopists (n = 4) evaluated and identified the papilla according to the Inomata classification using white light imaging and TXI. The degree of agreement with the evaluation of supervising physicians (*n* = 4) was examined using the McNemar test.

**Results:**

In the trainee group, the kappa coefficient agreements were κ = 0.346 and κ = 0.754 for white light imaging and TXI, respectively. When further evaluated, the separate and septal types of papilla groups showed an increased concordance rate in one of the four trainees (76.67%–96.67%, *p* = 0.031, respectively). Moreover, comparison for two‐group evaluation showed an increased kappa coefficient in two of four trainees (0.34–0.92, *p* = 0.010, 0.45–0.92, *p* = 0.024).

**Conclusions:**

Observation of the duodenal papilla using TXI improved papillary differentiation and suggested the potential of TXI as a clinical tool. Further study of this method is necessary; it is expected to help reduce cannulation time and the incidence of pancreatitis.

## INTRODUCTION

Over half a century since it was first reported by McCune et al. in 1966, endoscopic retrograde cholangiopancreatography (ERCP) still plays an important role in the diagnosis and treatment of biliopancreatic disease.^1^ Transpapillary biliary cannulation is difficult, especially for inexperienced endoscopists; hence, the rate of biliary cannulation is low. Biliary cannulation methods can be broadly categorized into either wire‐guided or contrast‐based techniques, each of which has variations in insertion methods and is subject to numerous discussions.^2^

A recent meta‐analysis showed that the success rate of biliary cannulation was 89.3% (95% confidence interval [CI] 0.866–0.919), indicating a significant number of failures.^3^ Furthermore, ERCP procedures conducted at hospitals with smaller ERCP volumes have a significantly higher rate of adverse events.^4^ Therefore, providing appropriate guidance and education for inexperienced endoscopists with relatively little ERCP experience is important for high‐volume centers.

There are two points for smooth biliary cannulation: 1) papillary opening, and 2) intrapapillary bile duct. In Japan, the importance of identifying the structure of the papilla of Vater opening, which is considered the first barrier to successful biliary cannulation, has long been recognized.^5^ However, insufficient attention has been paid to recognizing the morphology of the papilla, which makes biliary cannulation difficult. In particular, it is difficult for trainees to understand the papillary morphology accurately. We believe that accurate recognition of the morphology of the bile duct opening is an essential factor determining the success of bile duct cannulation.

**FIGURE 1:**
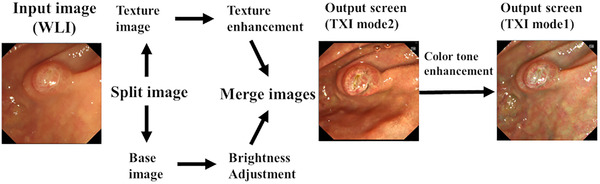
Algorithm for texture and color enhancement imaging. This algorithm outputs two types of texture and color enhancement images: mode2, which enhances texture and brightness, and mode1, which enhances texture, brightness, and color tone.

Texture and color enhancement imaging (TXI) is an imaging technique that optimizes three mucosal surface elements: structure, color, and brightness. It has been reported that slight changes in color tone and structure in images that are difficult to observe using white light imaging (WLI) can be enhanced using TXI; this enhancement contributes to an improved observation of lesions (Figure 1).

TXI mode2 enhances structure and brightness, while TXI mode1 is optimized to enhance structure, brightness, and color tone. TXI mode1 has been reported to be superior to WLI in the detection of early gastric cancer.^6^ Moreover, TXI mode1 can enhance color changes and improve the visibility of lesions suggestive of squamous cell carcinoma in the pharynx and esophagus, compared to WLI.^7^ Therefore, we aimed to investigate whether trainees inexperienced in ERCP could improve their understanding of the papilla of Vater morphology using TXI mode1.

## METHODS

### Study design and ethical considerations

This retrospective single‐center study was conducted in Japan and used electronic medical records to extract data regarding participants' age, sex, and the endoscopic procedure they underwent. All patients received detailed information and provided informed consent before undergoing the ERCP procedure. Informed consent for the study was obtained using the opt‐out approach, with details being posted on the website of the institutional review board. This study was approved by the Institutional Review Board of Saitama Medical University International Medical Center (2021–203).

### Patients

**FIGURE 2:**
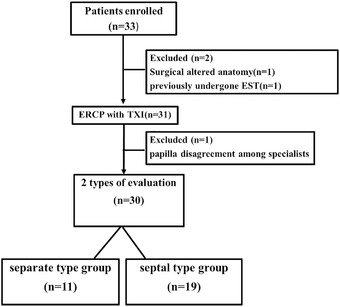
Enrollment flow chart We evaluated the procedure of biliary cannulation according to the *Inomata* classification (six types of evaluation) and divided papilla into two groups to determine their distinctive qualities: **Separate type group** and **Septal type group**. **Separate type groups**: Papilla₋I (***Betsukaiko***) and Papilla‐G (***Tamanegi***). **Septal type groups**: Papilla‐A (***Kesetsu***), Papilla‐U (***Heitan***) Papilla‐V (***Jyumo***), and Papilla‐LO (***Tatenaga*)**.

A total of 33 patients aged ≥20 years undergoing ERCP sessions at Saitama Medical University International Medical Center for bile duct stones, pancreatic cancer, and bile duct cancer were enrolled between September 1, 2021, and December 28, 2021. We excluded patients with surgically altered bowel anatomy and those who had previously undergone endoscopic sphincterotomy (Figure 2).

### Procedure

TJF‐290V duodenoscopes were used (Olympus Medical Systems, Tokyo, Japan). A conventional ERCP catheter (S01‐20‐70‐1; MTW Endoskopie Manufaktur, Wesel, Germany) was used for the biliary cannulation and injection of contrast media. Our institution's protocol required physicians to attempt biliary cannulation first using conventional contrast cannulation. A 0.025‐inch guidewire (G‐240‐2545A, Visiglide2; Olympus Medical Systems) was used for contrast cannulation. If cannulation was difficult to perform using this first method, we used the pancreatic duct guidewire placement method as a second option.

Using the classification method of papilla pattern proposed by Inomata,^5^ we compared the agreement rate between the trainees' answers and experts' answers on the papilla of Vater using TXI mode1.

As shown in Figure 1, mode1 is formed from mode2, and mode1 is more color‐enhanced than mode2. TXI mode1 differs more from WLI than does TXI mode2; it also emphasizes the papillary structure more. Therefore, TXI mode1 was adopted in this study.

After inserting WLI and obtaining the frontal view of the papilla, the papillary area was observed for 20 s, followed by observation using the TXI mode1 for 20 s. After the papilla was recognized, cannulation was performed by the trainee using the TXI mode1 in all cases, and the papillary pattern recognized using WLI and TXI was noted.

Experienced endoscopists who were present did not instruct on the morphology of the bile duct opening during the procedure. Trainees performed each ERCP; if successful biliary cannulation of the bile duct could not be achieved within 20 min after starting the procedure, an experienced endoscopist took over the procedure. The video taken during each ERCP procedure was separated by WLI and TXI time periods. The video was then randomized, and each of the four trainees evaluated the papilla.

### Recruitment of endoscopists

The endoscopists selected to perform ERCP first were inexperienced endoscopists with less than 100 cases’ and less than three years’ experience performing ERCP. However, they had experience with at least 50 ERCP cases during which they received expert guidance during and after the procedure with feedback. Moreover, they had also observed at least 100 procedures performed by others, including experts, and discussed the Inomata classification each time. The ERCP experience of the selected trainees was three months, six months, one year, and three years for Trainees A, B, C, and D, respectively. Endoscopists with >10 years of experience and >1000 procedures were considered experienced.

### Classification of the Vater papilla

According to the Inomata classification,^5^ the papilla of Vater was classified into one of six categories. We classified the papillae into two groups to determine their distinctive qualities: the separate and septal type groups. We divided the papilla of Vater into separate and septal groups since the method of biliary cannulation differs greatly between the two groups.

Separate papillae were defined as those with a recognizable orifice, and septal papillae were defined as those with a confluence of the bile and pancreatic ducts.

**FIGURE 3:**
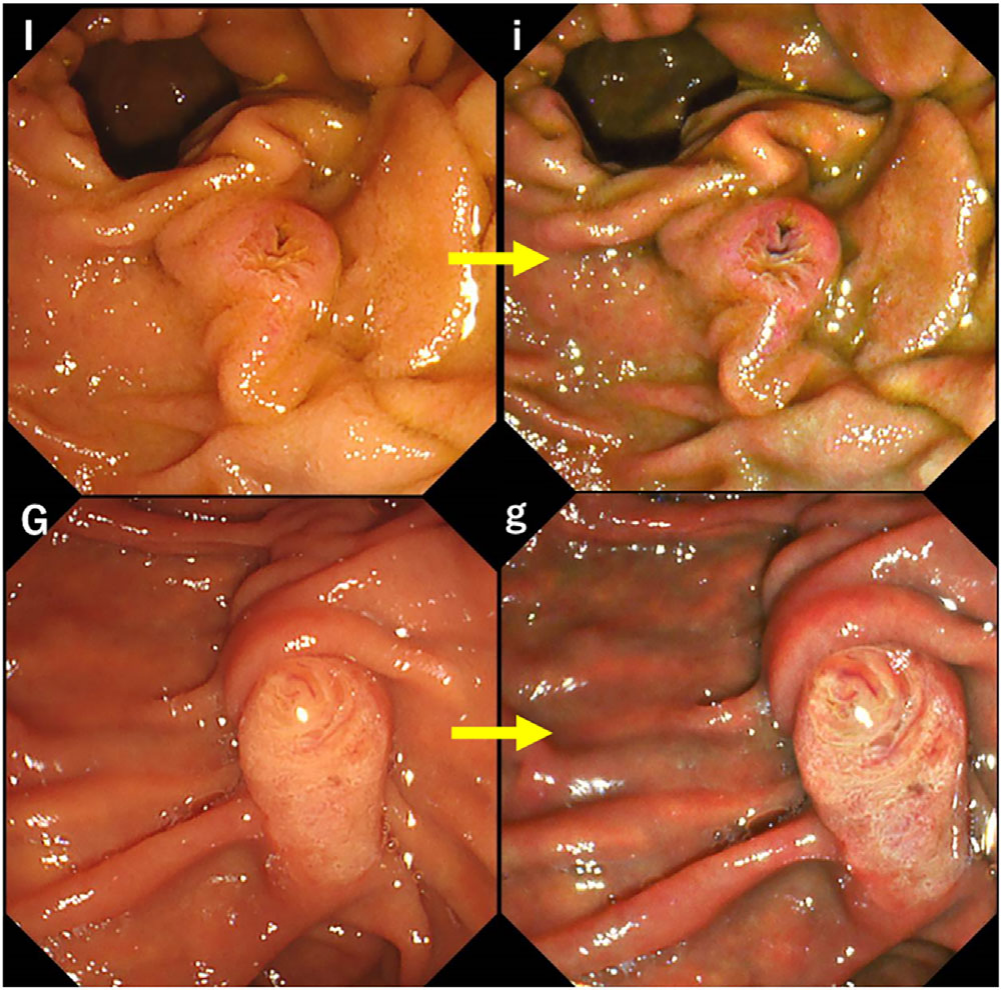
Separate type groups (Papilla‐I, G) Upper row (I: white light imaging, i: texture and color enhancement imaging mode1); Papilla‐I Lower row (G: white light imaging, g: texture and color enhancement imaging mode1) ; Papilla‐G

The papilla of Vater patterns in these two groups were further classified into one of the following six types:

**Separate type groups**: 1) Papilla‐I, comprising two separate, isolated orifices of the biliary and pancreatic ducts, with the opening on the oral or left side of the biliary duct and on the anal or right side of the pancreatic duct (Individual; ***Betsukaiko***); and 2) Papilla‐G, comprising gyrate, separate onion ducts. (Gyrate; ***Tamanegi***; Figure 3).

**FIGURE 4:**
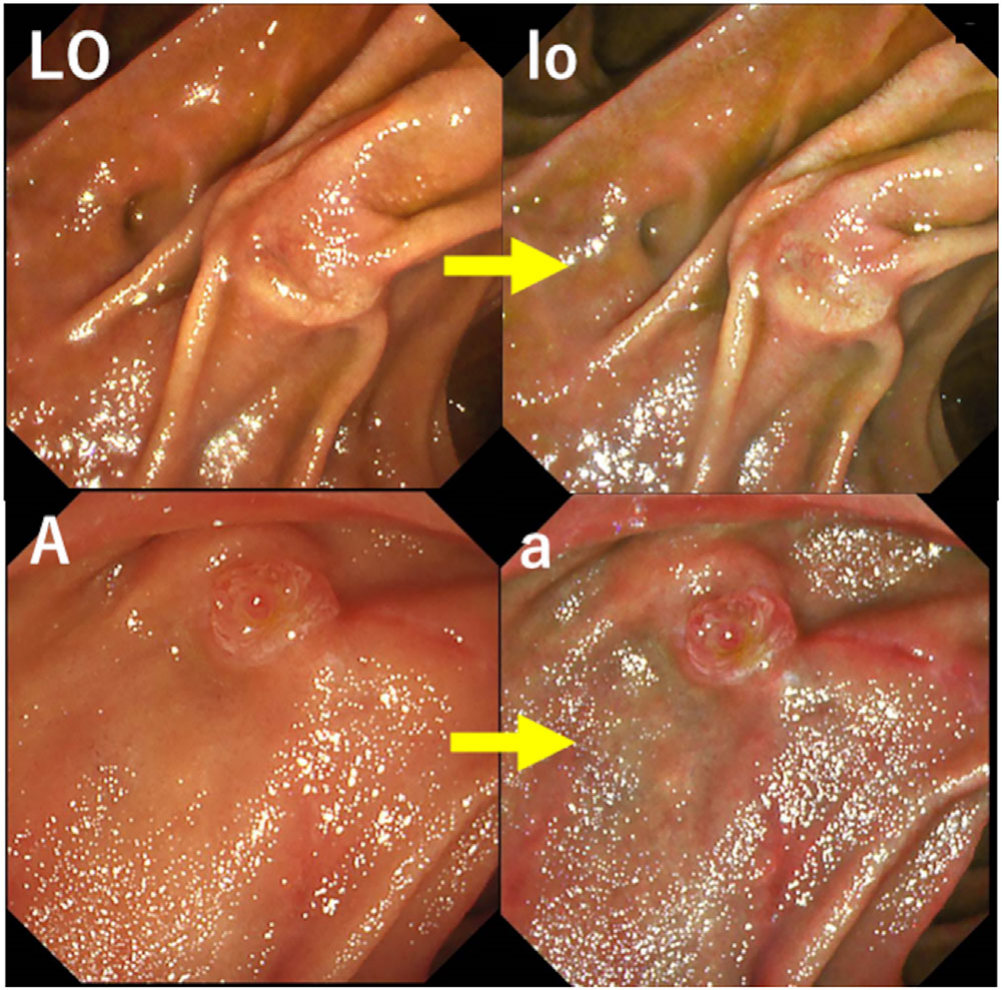
Septal type groups (Papilla‐LO, A) Upper row (LO: white light imaging; lo: texture and color enhancement imaging mode1); Papilla‐LO Lower row (A: white light imaging; a: texture and color enhancement imaging mode1); Papilla‐A

**FIGURE 5:**
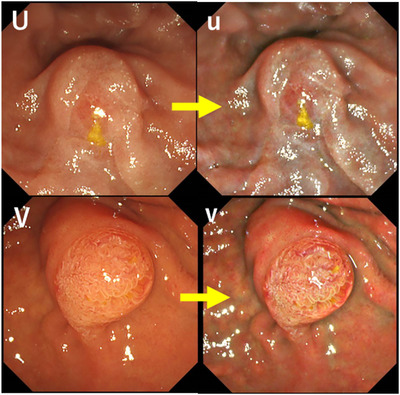
Septal type groups (Papilla‐U, V) Upper row (U: white light imaging; lo, u: texture and color enhancement imaging mode1); Papilla‐U Lower row (V: white light imaging; v: texture and colour enhancement imaging mode1); Papilla‐V

**Septal type groups**: 3) Papilla‐A, comprising a typical papilla with an annular shape, with some having nodular changes on the oral side of the center (10–11 o'clock) and others for which these features were difficult to discern (Annular; ***Kesetsu***); 4) Papilla‐U, unstructured without a clear orifice (Unstructured; ***Heitan***); 5) Papilla‐V, comprising a villous papilla with an almost even choriocapillaris shape across the opening (Villous; ***Jyumo***); and 6) Papilla‐LO, comprising longitudinal grooves continuous with the orifice, with the length of the grooves being longer than the transverse diameter of the biliary duct axis of the papilla (Longitudinal; ***Tatenaga***; Figures 4 and 5).

### Definitions and measurement of outcomes

**Definition of correct answer**: Four experienced endoscopists discussed the correct answer for the papillary type. The type of papilla they agreed upon was considered the correct answer for both WLI and TXI. If at least three of them disagreed, the papilla was considered unclassifiable and excluded from the analysis.

**Primary and secondary endpoints**: The primary endpoint was evaluation of the recognition of papillary morphology by trainees using TXI and the agreement rate with experienced endoscopists’ recognition. The secondary endpoint was biliary cannulation rate.

### Statistical analysis

The concordance rate and coefficient of agreement for each trainee (A–D) with the expert's matching response (two‐group evaluation) were calculated for each method. The concordance rate was calculated as the percentage of coinciding trainee and expert judgments. The coefficient of agreement was evaluated using Cohen's kappa coefficient. McNemar's test was used to compare the agreement rates between the WLI and TXI methods. Inter‐method comparison tests were also performed on kappa coefficients. The inter‐rater agreement of four experts or four trainee raters was calculated for each test judgment. The inter‐rater agreement was evaluated using the Fleiss' kappa value.

The secondary endpoint, biliary cannulation rate, was calculated as the percentage of cases with successful biliary cannulation and its 95% CI.

A two‐sided *p*‐value <0.05 was considered statistically significant. Statistical analysis was performed using SPSS version 26.0 for Windows (IBM Japan, Tokyo, Japan).

## RESULTS

A total of 31 patients were included; nine with common bile duct stones, 10 with pancreatic cancer, three with intrahepatic bile duct cancer, and nine with distal bile duct cancer. When evaluating the two groups of papilla, the concordance rates and the kappa coefficient for each trainee (A–D) were compared with the experts’ matched responses. In 30 cases, the experienced physicians agreed upon the classification of papilla into one of the six defined types, and the one case in which they disagreed was excluded.

The separate and septal type groups included 11 and 19 patients, respectively. Papilla‐I, Papilla‐G, Papilla‐A, Papilla‐V, Papilla‐U, and Papilla‐LO were observed in 13.3% (4/30), 23.3% (7/30), 33.3% (10/30), 6.6% (2/30), 6.6% (2/30), and 16.6% (5/30) of the patients, respectively.

The concordance rate between the four trainees was evaluated, and Fleiss' kappa values were calculated for inter‐subject coefficient agreement. The two groups (separate group vs. septal group) of papilla agreements were evaluated, and the result for WLI was low (κ = 0.346), whereas the result for TXI was high (κ = 0.754). (Table 1).

**TABLE 1:** Agreement among the four raters in the trainee

				Inter‐subject agreement			
Evaluation	Mode	Endoscopists	n	Kappa^a^	95% CI^‡^		
Two groups of papilla of Vater^b^							
	WLI^c^						
		Trainee	4	0.346	0.202	,	0.489
	TXI^d^						
		Trainee	4	0.754	0.610	,	0.898

^a^ Kappa: Fleiss' kappa; ^‡^95 % CI: 95 % confidence interval

^b^ 2 groups of papilla: 0_[I, G]; 1_[A, V, U, LO]

^c^ WLI: White‐light imaging

^d^ TXI: Texture and color enhancement imaging

Comparison of the response concordance rate for the two‐step evaluation between WLI and TXI showed that Trainee A had a significant increase in concordance from 76.67% to 93.33% (*p* = 0.031). Furthermore, in the comparison test for the kappa coefficient, Trainee A showed a significant increase from 0.34 to 0.92 (*p* = 0.010), while Trainee C showed a significant increase from 0.45 to 0.92 (*p* = 0.024; Table 2, 2). The completion rate of the procedure by the trainee with TXI in 31 patients was 64.52% (95% CI 45.37–80.77), and the final biliary cannulation rate was 96.77% (95% CI 83.30–99.92; Table 3).

**TABLE 2:** (a) Inter‐method comparison of agreement rates for two‐groups evaluation

	Match rate								
	WLI^b^				TXI^c^				*p*‐value^a^
	%	95% CI^d^			%	95% CI			WLI vs. TXI
Trainee A	76.67	57.72	,	90.07	96.67	82.78	,	99.92	**0.031**
Trainee B	86.67	69.28	,	96.24	93.33	77.93	,	99.18	0.625
Trainee C	80.00	61.43	,	92.29	96.67	82.78	,	99.92	0.125
Trainee D	83.33	65.28	,	94.36	93.33	77.93	,	99.18	0.375

^a^ *p*‐value: McNemar test

^b^ WLI: White‐light imaging

^c^ TXI: Texture and color enhancement imaging

^d^ 95% CI: 95% confidence interval

Match rate: Percentage of judgments in agreement

*n* = 30 (Cases in which uniform answers were obtained from the experts)

**TABLE 2:** (b) Comparison of kappa coefficients for two‐groups evaluation between methods

	Kappa coefficient								
	WLI^b^				TXI^c^				
	κ^d^	95% CI^e^			κ	95% CI			*p*‐value^a^
Trainee A	0.34	−0.02	,	0.70	0.92	0.78	,	1.00	**0.010**
Trainee B	0.68	0.40	,	0.97	0.86	0.67	,	1.00	0.320
Trainee C	0.45	0.10	,	0.81	0.92	0.78	,	1.00	**0.024**
Trainee D	0.53	0.20	,	0.86	0.85	0.66	,	1.00	0.147

^a^ *p*‐value: Kappa coefficient comparison test

^b^ WLI: White ‐light imaging

^c^ TXI: Texture and color enhancement imaging

^d^ κ: Cohen's kappa coefficient

^e^ 95% CI: 95% confidence interval

*n* = 30 (Cases in which uniform answers were obtained from experts)

**TABLE 3:** Endoscopic retrograde cholangiopancreatography (ERCP) with texture and color enhancement imaging (TXI)

	Total					
	*n*	*n*	%	95% CI		
Cannulation success rate	31	30	96.77	83.30	,	99.92
No trainee change rate	31	20	64.52	45.37	,	80.77

Abbreviations: CI, confidence interval; ERCP, endoscopic retrograde cholangiopancreatography; TXI, texture and color enhancement imaging.

## DISCUSSION

The difficulty of bile duct cannulation reportedly varies with the type of papilla.^8^ Moreover, accurate morphology of the papilla is an important factor for successful bile duct cannulation. This study investigated whether the rate of papillary recognition by trainee endoscopists could be improved by highlighting the contour of the papilla using TXI. We proposed using TXI, an image correction technique, by which trainees could improve the recognition of papillary morphology and the rate of biliary cannulation. TXI aims to bring out the subtle differences in tissue by emphasizing three image elements (texture, brightness, and color) in WLI. Ishikawa et al. suggested that the overall color differences were more pronounced using TXI^6^; furthermore, Abe et al. reported that TXI mode1 were superior to WLI in detecting early gastric cancer.^9^

The strategies for biliary cannulation differ according to the papillary morphology. In cases of a separate orifice, selective cannulation can easily be performed by pushing the catheter against the orifice; TXI clarifies the location of the orifice and makes selective cannulation easier. TXI also makes the papilla slit more visible, which is expected to reduce instances of papillary misrecognition.

In nodal type papilla (Papilla‐A), the confluence of the bile and pancreatic ducts is almost always septal. TXI alone is not useful for biliary cannulation in Papilla‐A, even if the orifice is made clearer. It is difficult to distinguish between the septate and common ductal papilla using TXI alone. Papilla‐V, the choriocapillaris type, has an almost uniform choriocapillaris shape over the entire orifice. Most longitudinal (LO) papilla are septate; like with Papilla‐A, it is difficult to say that TXI increases the rate of biliary cannulation for Papilla‐LO.

Separate type groups (Papilla‐I and G) are reported to account for 30% of all cases, thus constituting a significant portion.^5^ In any case, it is important to distinguish between the separate type (Papilla‐I and G) and septal type groups (Papilla‐A, V, U, and LO), given that they warrant different approaches to biliary cannulation.

Similar to the morphology and type of papilla, the oral protrusion of the papilla is also strongly correlated with the difficulty of biliary cannulation. Watanabe et al. performed logistic regression analysis with bile duct cannulation difficulty as the target variable in patients who underwent ERCP by experienced endoscopists.^10^ Their study identified Protrusion‐L as a significant risk factor [OR 2.956; 95% CI 1.115–7.84; *p* = 0.029], which was further confirmed as an independent risk factor on multivariate analysis.

Misrecognition of the papillary morphology can result in an incorrect approach and a time‐consuming cannulation process. In fact, it is common to spend more time on papilla that are relatively less difficult to cannulate, such as the separate (Papilla‐I) and onion (Papilla‐G) types. For the papillary pattern, the first attempt by an inexperienced endoscopist in all patients required significantly more attempts (Papilla‐A, *p* = 0.028; ‐LO, *p* = 0.028; and ‐G, *p* = 0.033); even in the onion type (G), where the bile duct and pancreatic duct are separate.

With TXI, it may be possible to reduce the number of cases in which separate type groups (Papilla‐I and G) are misidentified as septal type groups (Papilla‐A, V, U, and LO). TXI may also lower the number of attempts at biliary cannulation.

The time taken for cannulation and the imaging rate of the pancreatic duct are risk factors for post‐ERCP pancreatitis.^11^ Therefore, the type of the papilla should be correctly recognized for a smooth undertaking of the procedure. Since even experts may encounter difficulty un accurately classifying papilla according to the Inomata classification, the correct papillary type was decided after discussion among the experts in this study. Given that experienced experts may have different opinions as to the papillary type, the agreement rate increased even among them when TXI was used (κ = 0.523–0.812).

Among the inexperienced endoscopists, Trainees A, B, C, and D had three months, six months, one year, and three years of ERCP experience, respectively. Trainees A and C significantly improved their recognition ability by using TXI in the two‐group evaluation; their ability was almost equal to the papillary recognition ability of experts. Although there was a sharp increase in κ value when Trainee A used TXI (κ = 0.34–0.92), there was no other notable feature, except that Trainee A had lesser experience than the other trainees. It is therefore very difficult to determine the reason underlying this sharp increase in κ value. Trainee D also showed improved papillary recognition, although the difference was non‐significant. Although Trainees A, B, and C, who were the most inexperienced, had a low agreement rate with experts in WLI, their papillary recognition ability was close to that of the experienced endoscopists when using TXI. Hence, if a trainee has a certain level of experience, their ability to recognize papilla can be compared to that of experienced endoscopists, even without the use of TXI.

Our results indicate that proposed biliary cannulation using TXI can be a clinical tool for improving papillary differentiation. TXI can greatly emphasize the differences in the color tone of the fine mucosal surface of the papilla. Moreover, TXI can selectively emphasize the irregularities of the papilla and highlight subtle tissue differences, such as the mouth of the bile duct and the pancreatic duct, and slight changes in morphology and color.

This study has some limitations, including its single‐center design, the low number of trainees, and differences in the experience levels of trainees. In addition, the sample size was small since this was a pilot study. Moreover, although six types of papilla were reported in the original Inomata classification, we investigated papilla divided into two major types in this study.

In conclusion, TXI may improve the ability of endoscopists to identify the papilla of Vater. Although more cases need to be accumulated, TXI may help inexperienced endoscopists perform biliary cannulation.

## CONFLICT OF INTEREST

The authors declare that there are no conflicts of interest regarding the publication of this paper.

## Data Availability

The data used to support the findings of this study are available from the corresponding author upon reasonable request.
